# A decade of anti-IL-1 therapy in CAPS - a spectrum of efficacy in this spectrum of diseases

**DOI:** 10.1186/1546-0096-13-S1-O65

**Published:** 2015-09-28

**Authors:** T Lane, RG Williams, DM Rowczenio, T Youngstein, H Trojer, RJ Pepper, PA Brogan, PN Hawkins, HJ Lachmann

**Affiliations:** 1 University College London, National Amyloidosis Centre, Division of Medicine, London, UK; 2 University College London, Paediatric and Adolescent Rheumatology, Institute of Child Health, London, UK

## Introduction

Discovery of the role of the IL-1 inflammasome in CAPS has revolutionised treatment, and anti-IL-1 therapies have successfully switched off disease activity in many patients. More than 110 CAPS patients (including 24 children) in the UK have been treated with drugs targeting IL-1, over 90% of these have had complete resolution of disease.

## Objectives

We describe a cohort of 10 patients who had sub-optimal response to canakinumab and/or anakinra.

## Patients and methods

Patients were diagnosed by genetic sequencing and clinical assessment. Serial SAA concentrations were analysed, along with patient reported symptom scores. Partial response was defined as improved but incomplete resolution of patient-reported symptoms and/or reduction, but not normalisation, of SAA concentration.

## Results

Patients 1 (male; age at treatment: 40; mutation in *NLRP3*: T436I; phenotype: CINCA) and 2 (female; 43; R260W; MWS) experienced modest and transient reductions of SAA and symptoms when treated with canakinumab. Both patients developed marked inflammation with morphea-like rash shortly afterwards. Both were switched to anakinra with excellent effect.

Patient 3 (male; 47; A439V; FCAS) weighed 130kg, had a partial response to both 300mg canakinumab q8w and 200mg anakinra daily. Patients 4 (female; 1; Y570F; CINCA), 5 (male; 16; S547C heterozygote; CINCA) and 6 (female; 59; Y547C mosaic; atypical) had severe disease. Patient 4, experienced partial response to canakinumab and anakinra, although feels better on 300 mg canakinumab (10mg/kg q8w). Patient 5 has not responded to 10mg/kg q8w canakinumab started 12 weeks ago. Patient 6 has been treated with both canakinumab and anakinra over 10 years, at times concurrently. She experienced a severe flare with aseptic meningitis after attempted conversion to canakinumab. She is now on anakinra 300mg daily and inflammation and headaches have remained consistent.

Patients 7 to 10 had CNS inflammation symptomatically, and on lumbar puncture/MRI. Patients 7 (male; 41; T348M; CINCA) and 8 (female; 20; A352T; CINCA) had resolution of most CAPS symptoms and SAA normalised on canakinumab 300mg q8w, however headaches and fatigue continued. Both had previous strokes attributed to CNS inflammation. Patient 7 had some improvement on anakinra. Patient 9 (male; 48; E567K mosaic; atypical), weighing 102kg, had complete resolution of most CAPS symptoms and SAA normalised, but headaches, poor balance and fatigue continued on both treatments. Patient 10 (male; 24; T348M; MWS) had a complete normalisation of inflammatory markers and peripheral symptoms on 300mg canakinumab. However, headaches and fatigue remained. Encouragingly, in patients 9 and 10 fatigue and mood have improved after over 5 years of treatment.

## Conclusion

Patients 1 and 2 raise the possibility of IL-1α mediated inflammation. Patients 3-6 suggest incomplete blockade of IL-1 activity with the maximum doses available on the NHS. Finally in patients 7-10 who had decades of CNS inflammation prior to treatment, headache and fatigue linger.

